# Detecting model misconducts in decentralized healthcare federated learning

**DOI:** 10.1016/j.ijmedinf.2021.104658

**Published:** 2021-12-09

**Authors:** Tsung-Ting Kuo, Anh Pham

**Affiliations:** UCSD Health Department of Biomedical Informatics, University of California San Diego, La Jolla, CA, USA

**Keywords:** Model Misconducts, Federated Learning, Predictive Modeling, Electronic Health Record, Blockchain Distributed Ledger Technology

## Abstract

**Background:**

To accelerate healthcare/genomic medicine research and facilitate quality improvement, researchers have started cross-institutional collaborations to use artificial intelligence on clinical/genomic data. However, there are real-world risks of incorrect models being submitted to the learning process, due to either unforeseen accidents or malicious intent. This may reduce the incentives for institutions to participate in the federated modeling consortium. Existing methods to deal with this “model misconduct” issue mainly focus on modifying the learning methods, and therefore are more specifically tied with the algorithm.

**Basic Procedures:**

In this paper, we aim at solving the problem in an algorithm-agnostic way by (1) designing a simulator to generate various types of model misconduct, (2) developing a framework to detect the model misconducts, and (3) providing a generalizable approach to identify model misconducts for federated learning. We considered the following three categories: Plagiarism, Fabrication, and Falsification, and then developed a detection framework with three components: Auditing, Coefficient, and Performance detectors, with greedy parameter tuning.

**Main Findings:**

We generated 10 types of misconducts from models learned on three datasets to evaluate our detection method. Our experiments showed high recall with low added computational cost. Our proposed detection method can best identify the misconduct on specific sites from any learning iteration, whereas it is more challenging to precisely detect misconducts for a specific site and at a specific iteration.

**Principal Conclusions:**

We anticipate our study can support the enhancement of the integrity and reliability of federated machine learning on genomic/healthcare data.

## 1. Background and significance

### 1.1. Introduction

To accelerate genomic medicine research and facilitate quality improvement, researchers have started cross-institutional collaborations for better uses of Machine Learning (ML) on genomic and healthcare data [1–2], especially for diseases/conditions with relatively rare samples. While health systems and hospitals may exchange patient-level records to increase sample size in a collaborative learning process, potential concerns such as the risk of re-identification can still be a burden for direct data sharing [3]. To protect privacy of patients, federated learning methods (or “data-private” collaborative learning [4]), were developed to only exchange aggregated ML models among institutions without disseminating patient-level data [5–6]. However, the central server which orchestrates the federated learning process may still pose a security risk of single-point-of-failure. To mitigate this problem, several decentralized methods have been proposed [7–13] based on blockchain technology [14–17]. Decentralized approaches adopt semi-honesty as the underlying adversary assumption. That is, each site would only submit correct models, and will not accidentally or intentionally make mistakes to submit incorrect models. Nevertheless, this assumption may be too optimistic in the real world. For example, a site may accidentally submit old models due to network latency, or worse, may be intruded or controlled by malicious users who submit fake models on purpose [18–19]. Such incidents whether “negligent” or “hacked” can lead to bioethical concerns, and thus reduce the incentives for institutions to participate in the federated modeling consortium. This exhibited precaution affirms the need for a proactive approach when it comes to data privacy protection, which asks for ML researchers to detect and patch vulnerabilities before such possible weaknesses are exploited [20].

### 1.2. Data misconduct

Given the current climate of cyber doubts and suspicions [21], it is prudent to propose defenses against possible dissemination of unauthentic information, whether at the level of data (“data misconduct”) or model (“model misconduct”). *Data* misconduct (i.e., input data tampering) [20,22–27] refers to the manipulation of training and/or testing data features, or training data labels [28–29] to alter classification efficacy, assuming the process of model construction itself is honest. Researchers have investigated data misconduct such as adversarial machine learning [22–23], where the attackers try to impact the model through “poisoning attacks” during the re-training process by providing malicious training data [26–27], or through “evasion attacks” on testing data that lead to misclassification [20]. In the particular field of federated ML, a related research topic is about the “backdoor attack” of transfer learning [24–25]. Suppose there are two sites *S_1_* and *S_2_*, and the transfer learning algorithm learns a model using data from *S_1_* and then transfers the model to *S_2_* (i.e., the model of *S_1_* is open/accessible while the model of *S_2_* is not). An attacker can try to create malicious test data as the input of the model of *S_2_* based on the understanding of the model of *S_1_*, aiming at obtaining misclassification results. Since the misconduct happens by manipulating the testing data, this attack also belongs to data misconduct. In short, with data misconduct the adversary can impact the model through *indirect* means of data injection (i.e., an attacker cannot update model parameters directly).

### 1.3. Model misconduct

On the other hand, *model* misconduct focuses on unauthentic rendering of model parameters from *honest* data, when the process of model construction is not guaranteed to be honest. That is, each participating learner has the *direct* capability to provide wrong models during the learning process. Based on the sheer size difference between data and models, the effort needed to influence model parameters directly is also substantially less than what would be required to infect input data. Therefore, model misconduct is an important issue that needs to be considered in the federated learning process. For *centralized* federated learning (i.e., data are stored locally but the exchanged models are handled by a central server), recent studies examined the “targeted” attacks (i.e., misclassification of one particular label) with the threat assumption of having one single malicious agent at a given time [30–31]. In case the attacker controls several data-contributing agents, the objective of the attack itself remains to be targeted, whether in single-shot or repeated attacks [32–33]. Existing literature also investigated “untargeted” attacks (i.e., not specifically aiming at influencing certain label) [34–35] such as impacting the global consensus modeling process to stop convergence, to increase error rate, and/or to degrade model performance, including a threat model with multiple bad-faith agents [36]. Within *fully-decentralized* approaches (i.e., without a trusted server), prior related studies centered on singular machine learning algorithms (e.g., Stochastic Gradient Descent [7–8]) and therefore are more specifically tied with the algorithm. When algorithms are allowed to vary, past approaches turn to either the underlying blockchain framework [37–38], the consensus protocol [39], relying on simple statistics [40] and/or node-specific performance [41–42] to detect anomalies, or providing incentives to increase trust [43].

## 2. Objective

To investigate the issue of model misconduct in decentralized modeling, we aim at (1) designing a simulator to generate various types of model misconduct, (2) developing a framework to detect the model misconducts without modifying the blockchain setting or the consensus protocol, and (3) providing a generalizable approach to identify model misconducts concerns for decentralized federated learning in an algorithm-agnostic way. Our novel framework aims to inherit the advantage of protecting patient privacy from known privacy-preserving federated learning designs [5–6], as well as the ability to resist single-point-of-failure thanks to the decentralized approach on a blockchain platform [14–17,44–46].

## 3. Materials and methods

### 3.1. Misconduct threat models and adversarial goals

We considered the following three categories inspired by “academic misconducts” of plagiarism, fabrication, and falsification, of which a site may try to hide their information, inspect information from other sites, or disturb the learning process. These misconduct categories are common yet only partially investigated in existing studies [7,8,41,42,47], while we summarized and explored all of them in our study: (a) *Model plagiarism*: a site becomes a free-rider and just submits a copy of a model in a previous learning iteration, trying to hide their own information while inspecting models from other sites. (b) *Model fabrication*: a site submits a mock-up model (e.g., assigning random values or just sending empty models with all zeroes), trying to hide information and disturb the ML process, making the model converge incorrectly or even never converge. (c) *Model falsification*: a site submits a model tweaked from their actual result, trying to influence the learning process. Under these three categories, we summarized 10 types of threat models and their adversarial goals (Table 1).

### 3.2. Data

We chose three datasets for their varied sample sizes and ratios of positive/control, and a relatively reasonable number of covariates. In the context of decision support in healthcare, a modest-sized model might be beneficial for human interpretation by physicians and researchers. The three datasets are as follows (all predicting binary outcomes): (1) Edinburg Myocardial Infarction (“Edin”) [48], a publicly-available dataset with 9 covariates and 1,253 samples, to predict the presence of disease (21.9% positive); (2) Cancer Biomarker (“CA”) [49], a public dataset with 2 covariates and 141 patients, to predict the presence of cancer (63.8% positive); (3) Clostridium Difficile Infection (“C-Diff”) [50], a dataset collected from the UCSD Health System with 25 covariates and 157,493 patients, to predict the presence of infection (1% positive). The Institutional Review Board (IRB) at UCSD approved this study (190385X) on August 14, 2020, with the informed consent requirement exempted. We simulated four participating sites by randomly splitting each dataset into four parts (25% of patient-level data), and randomly sampled 50% records (containing at least one positive and one negative sample) for model training. In addition, we performed all experiments in accordance with relevant guidelines and regulations.

### 3.3. Model and adversary Knowledge/Capability assumptions

We adopted GloreChain [10], a blockchain-based batch decentralized Logistic Regression algorithm, with the following hyper-parameters [11]: 1 s as the polling time period, 5 s as the waiting time period, 100 as the maximum per-level iteration, and 10^−6^ as the precision of the convergence criterion. We repeated the above training process for 30 trials and collected the partially-trained “local” models (including the gradient vector and the variance–covariance matrix) and the combined “global” models (the coefficient vector) for each learning iteration. The number of iterations including the initialization one for the three datasets are Edin = 246, CA = 238, and C-Diff = 885.

We considered the following assumptions about adversary knowledge/capability: each site can access (a) its own “local” patient-level data, (b) the global models (246, 238, and 885 models, for Edin, CA, and C-Diff datasets, respectively), and (c) the local models from all 4 sites (984, 952, and 3540 models, for Edin, CA, and C-Diff datasets, respectively). Each site can manipulate its own local models, as well as the global models updated by that site, and then share these tampered models with the other sites. On the other hand, each site cannot access the patient-level data on other sites, nor can they manipulate the local models or the learning process of other sites.

### 3.4. Misconduct generation

We generated the models on the datasets using a GloreChain. The GloreChain models disseminated among sites included both “local” ones (i.e., the partially-trained gradient vectors and the variance–covariance matrices) and the “global” ones (i.e., the combined coefficient vectors). To simulate the generation of misconducts, we only focused on the partially-trained local models, because manipulation of the global models can be easily detected by each site (simply combine the local models to compare with the received global one). We simulated 11 misconduct scenarios (10 types of misconduct plus one for “all 10 types”). To generate misconducts on the partially-trained models, we iterated through each of the models from each site (e.g., 3540 models for the C-Diff dataset), and applied the misconducts with a pre-defined probability (0.25 in our experiment). For the 10 scenarios with only one misconduct type each, we applied the specific one directly. For the scenario with all 10 types of misconducts, we randomly selected 1 out of the 10 types. The details of the misconducts are described in Appendix A.

### 3.5. Misconduct detection

**Fig. 1.**
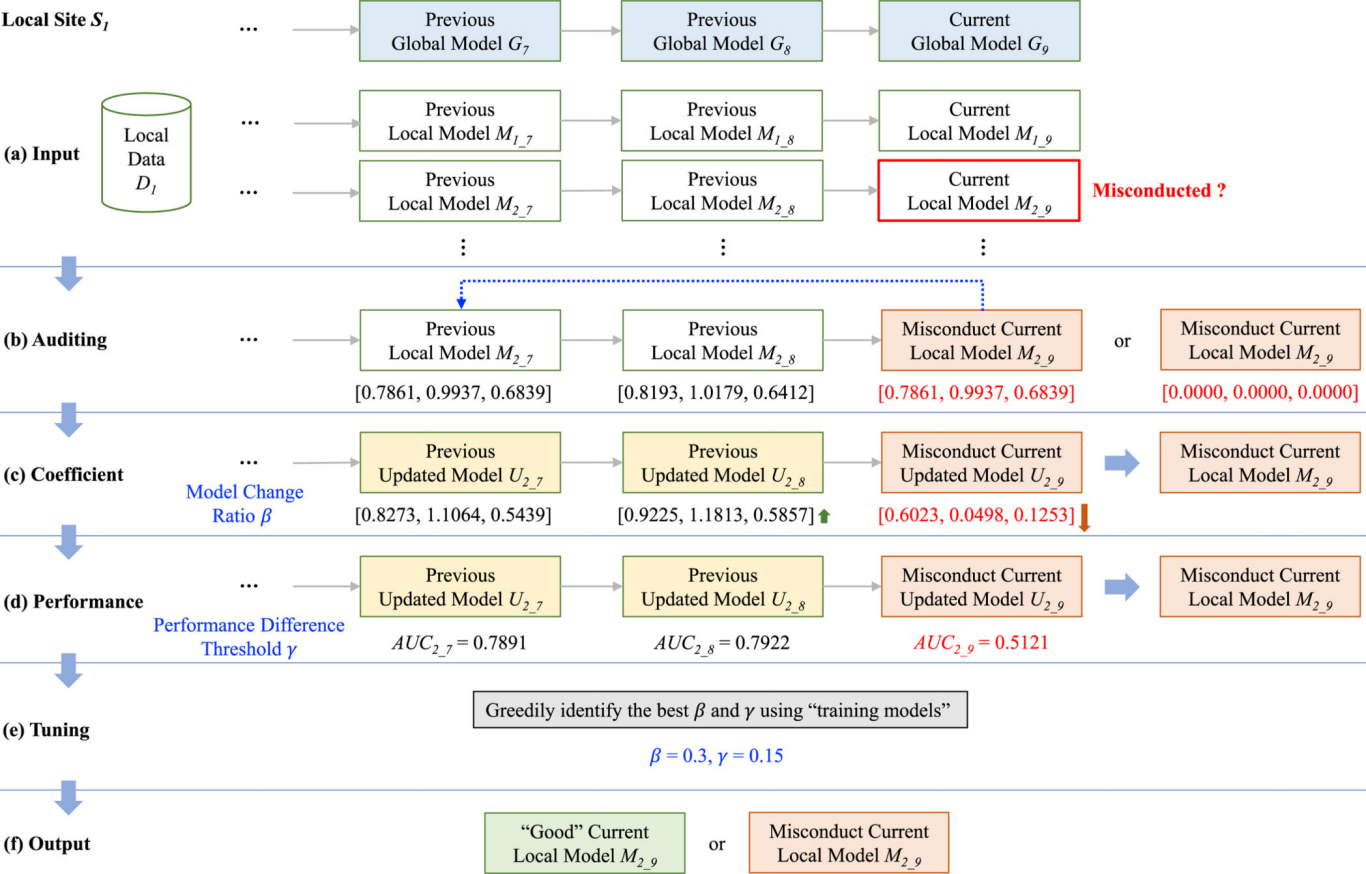
The model misconduct detection framework. In this example, suppose there are four participating sites in total, the current learning iteration is 9, and a site *S*_*1*_ would like to determine whether a newly received partially-trained model *M*_*2_9*_ commits misconduct. **(a)** The *input* includes *S*_*1*_′ s local patient-level data *D*_*1*_, as well as the global models (*G*_*1*_, *G*_*2*_, …, *G*_*9*_) and all local models (*M*_*1_1*_, *M*_*1_2*_, …, *M*_*4_9*_) in current and previous iterations. **(b)** The *Auditing* detector compares *M*_*2_9*_ with the historical local models and sees if it is copied from any of them and checks to see if *M*_*2_9*_ contains an empty gradient vector or variance–covariance matrix. **(c)** The *Coefficient* detector compares the updated model *U*_*2_9*_ (computed by updating *G*_*8*_, the global model of the previous iteration, using only *M*_*2_9*_) with the updated models in the previous iterations, to recognize any significant direction change (for example, the previous updated model has been increasing from *U*_*2_7*_ to *U*_*2_8*_, while the values drop suddenly from *U*_*2_8*_ to *U*_*2_9*_). **(d)** The *Performance* detector compares the evaluation result (e.g., computed using full Area Under the receiver operating characteristic Curve, or AUC [51,52], on *D*_*1*_) of the current updated model *U*_*2_9*_ with the average results of all previous ones (AUC of *U*_*2_1*_ to *U*_*2_8*_), and identifies any significant difference. **(e)** The tuning step finds the best the parameters β and γ using greedy search. **(f)** The *output* is a binary decision of whether *M*_*2_9*_ is misconducted. If none of the detectors pinpoint a misconduct, *M*_*2_9*_ is considered a non-misconducted model, otherwise a misconduct one.

To detect model misconducts from a site *K* at learning iteration *T* on a federated learning consortium with *N* participants, we developed a framework consisting of three detector components *Auditing*, *Coefficient*, and *Performance*, as shown in Fig. 1. The input for *K* includes its local patient-level data *D_K_*, as well as the global models (*G_1_*, *G_2_*, …, *G_T_*) and all local models (*M_1_1_*, *M_1_2_*, …, *M_N_T_*) from *N* sites in current and previous iterations (Fig. 1(a)). Each site cannot access the patient-level data from other sites to protect privacy, while all aggregated global/local models are shared and accessible for each site on the underlying blockchain ledger [9–13]. The output is a binary decision of whether *M_S_T_*, a local model disseminated by another site *S* at iteration *T*, is considered as a misconducted model or not (Fig. 1(f)). *M_S_T_* is considered a non-misconducted model if none of the detectors identifies potential misconducts; otherwise, it is considered “misconducted”. The three detector components, Auditing, Coefficient, and Performance, as well as the parameter tuning step, are described in Appendix B.

### 3.6. Experiment settings

**Fig. 2.**
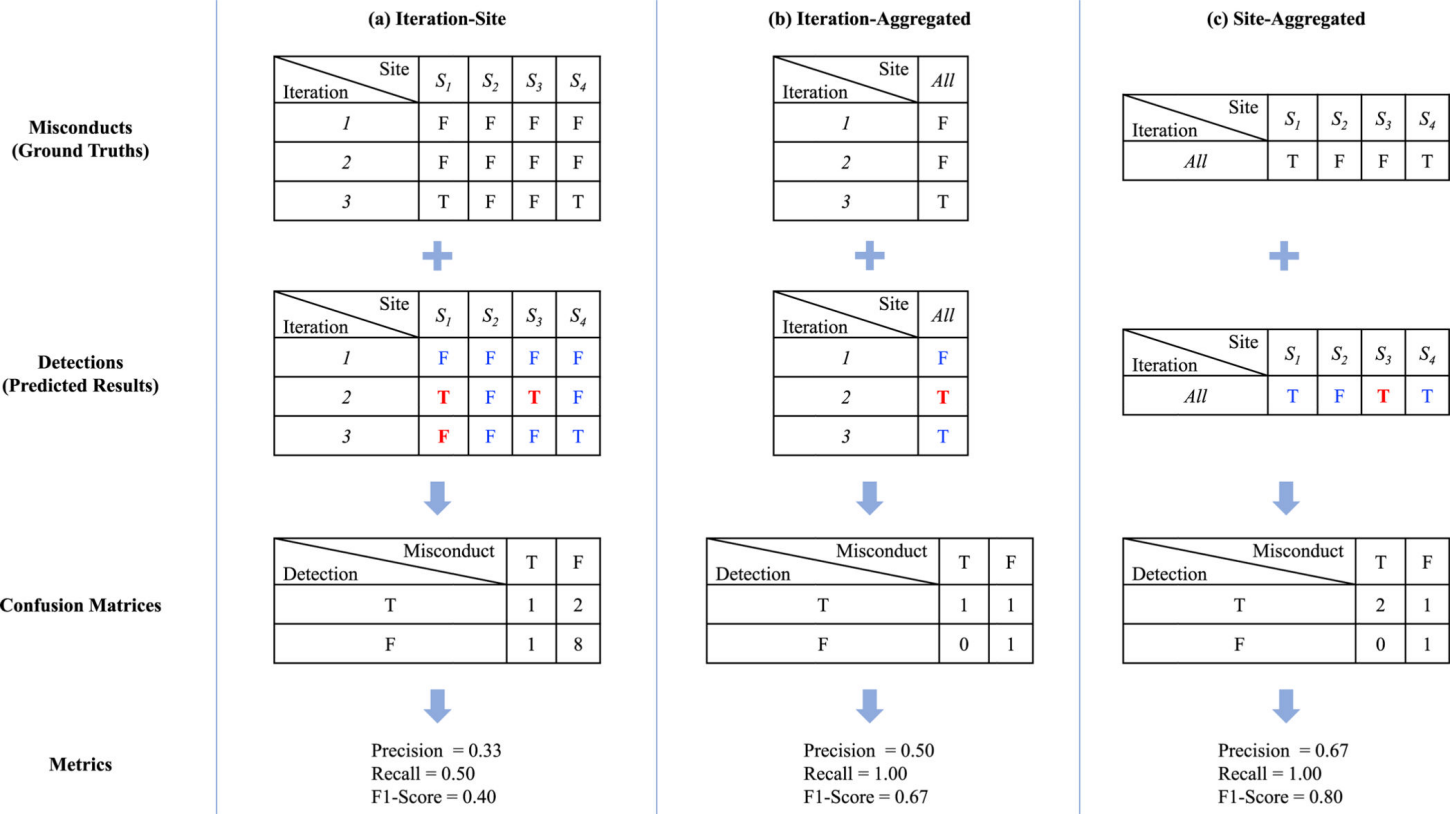
An example of the evaluation schemes of model misconduct detection. The misconducts, detections, confusion matrices, and metrics are shown in this 4-site, 3-learning-iteration example. A “T” indicates misconduct, and a “F” means no misconduct. The corrected detected results are in blue text, while the incorrect ones are in red and bold text. We adopted 3 different evaluation schemes to compute the metrics (i.e., precision, recall, and F1-score). **(a)** *Iteration-Site*. This scheme directly takes all 3 (learning iterations) * 4 (participating sites) = 12 ground truths and predicted results to compute the prediction, recall, and F1-score. **(b)** *Iteration-Aggregated*. This scheme first aggregates the results by learning iterations (rows) using “OR” operation (i.e., if in a learning iteration there is one misconduct on any site, it is considered as a misconduct learning iteration). Then, the aggregated values (i.e., the ground truths and predicted results for the three learning iterations) are used to compute the metrics. **(c)** *Site-Aggregated*. Just like Iteration-Aggregate, this scheme first aggregates the results by sites (columns) using “OR” operation, and then the aggregated values for four sites are used to calculate the metrics. (For interpretation of the references to colour in this figure legend, the reader is referred to the web version of this article.)

To evaluate the effectiveness of the detection, we adopted 3 evaluation schemes: *Iteration-Site*, *Iteration-Aggregated*, and *Site-Aggregated*, as shown in the example in Fig. 2. These three schemes correspond to different granularities of evaluation. For the Iteration-Site scheme (Fig. 2(a)), the misconducts needed to be detected exactly for a site in an iteration. Next, for Iteration-Aggregated (Fig. 2(b)), we focused on identifying the misconduct learning iteration, regardless of the site on which the misconduct happens. Finally, for Site-Aggregated (Fig. 2(c)), the main consideration was to recognize the misconduct on certain sites from any learning iteration. For all these three schemes, we calculated the precision, recall, and F1-score as our evaluation metrics. To validate the detection correctness of our detection method, we adopted a pertrial 10-fold cross-validation (CV). The details of CV and our implementation are described in Appendix C.

## 4. Results

### 4.1. Detection correctness

**Fig. 3.**
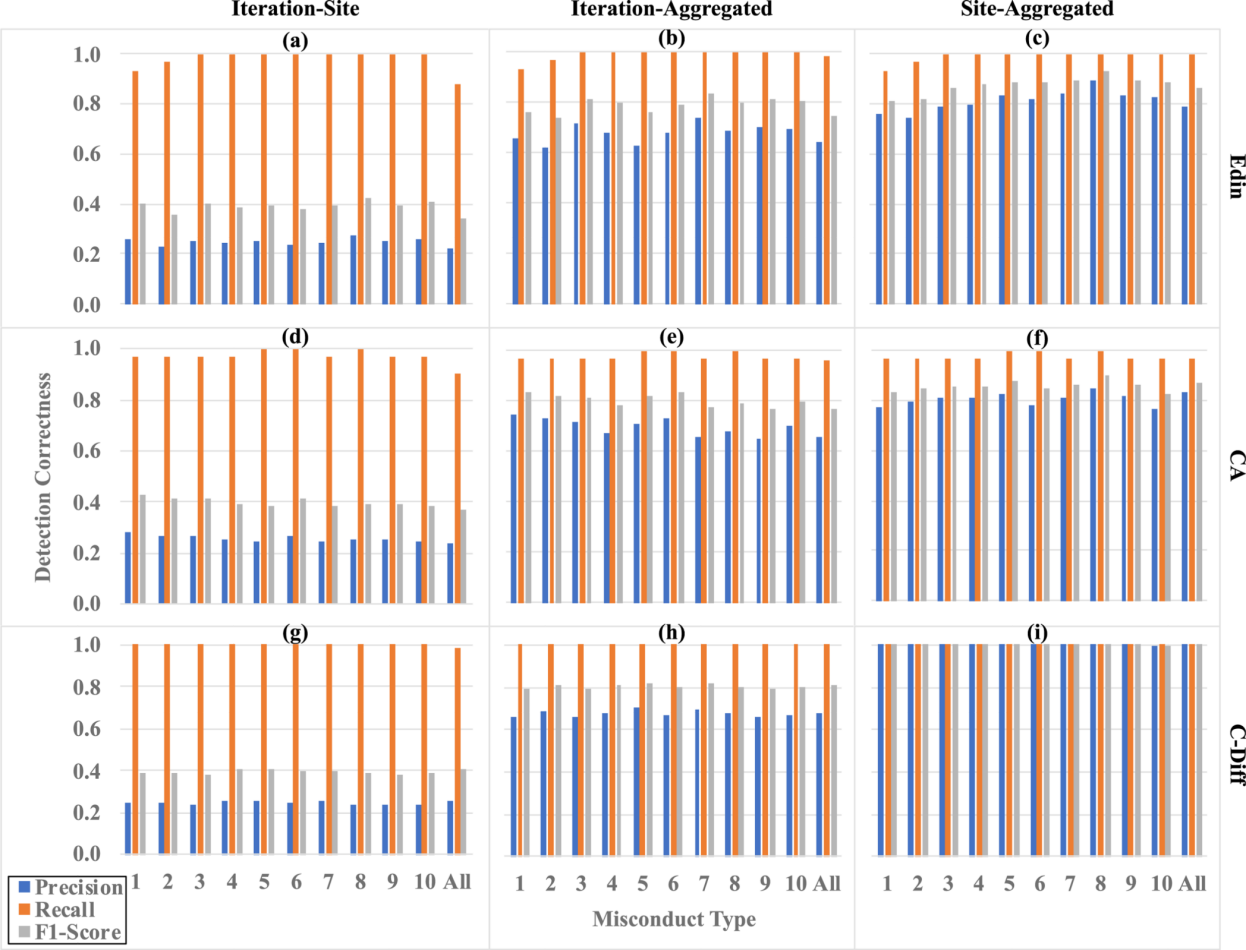
Detection correctness results for three datasets Edin, CA, C-Diff under three evaluation schemes Iteration-Site, Iteration-Aggregated, and Site-Aggregated. X-axes are the 11 misconduct types (10 single-type + 1 all-type), and Y-axes are the metrics of prediction, recall, and F1-score.

The correctness results of misconduct detection are illustrated in Fig. 3. In general, the recall is high (between 0.87 and 1.00) across different datasets, evaluation schemes, and types of misconducts. For the Iteration-Site evaluation scheme, the precision values are between 0.20 and 0.30, resulting in F1-scores around 0.40. For Iteration-Aggregated, the precisions fall between 0.62 and 0.75 and the F1-scores are between 0.73 and 0.84. For Site-Aggregated, the precision values are between 0.74 and 0.90 (resulting in F1-scores from 0.81 to 0.94) for the Edin and CA datasets, and nearly perfect (i.e., all 1.00 for precision and F1-scores) for the C-Diff dataset.

### 4.2. Ablation study

**Fig. 4.**
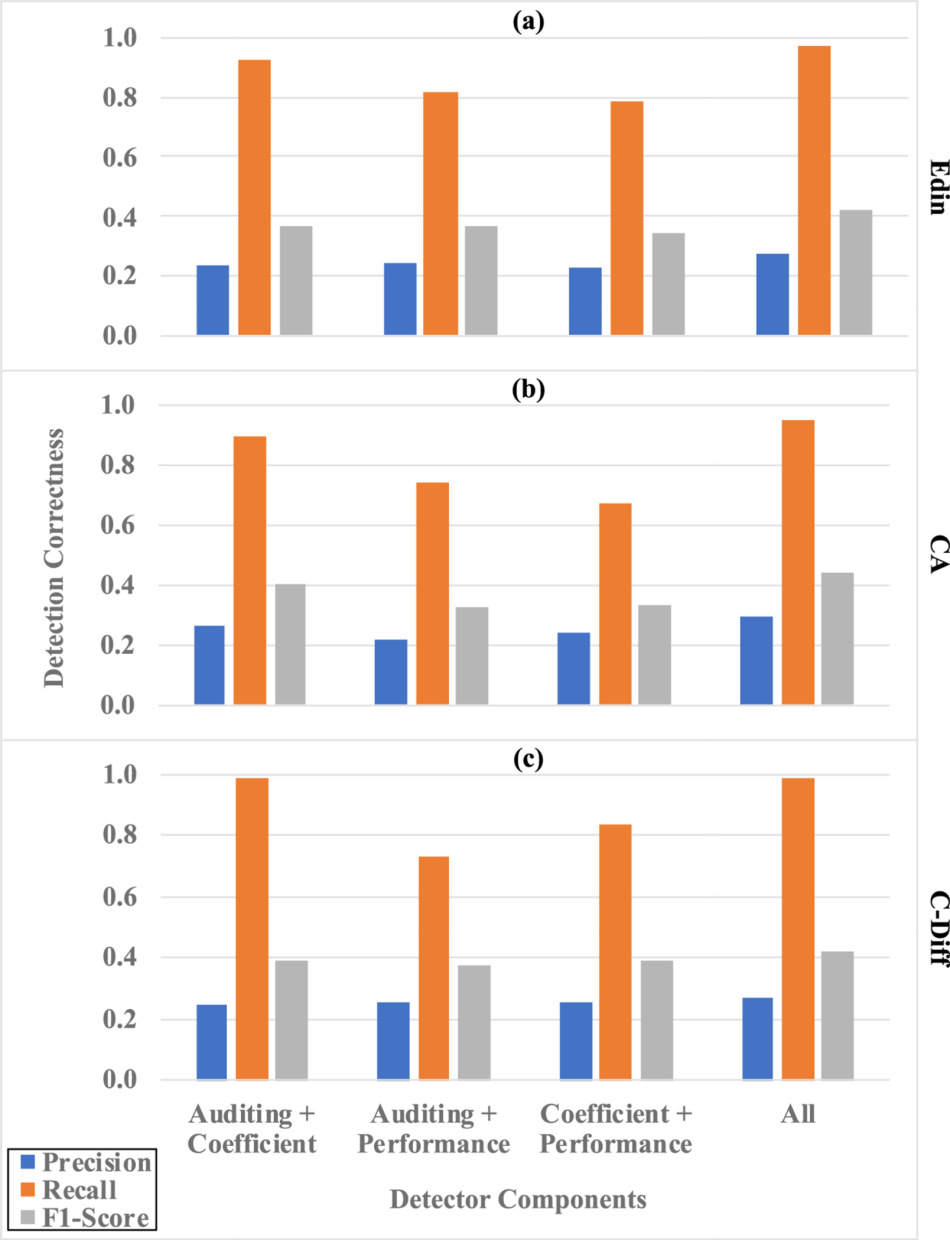
Ablation study results for three datasets Edin, CA, C-Diff under the Iteration-Site evaluation scheme. X-axes are the combinations of the three detector components, and Y-axes are the metrics of prediction, recall, and F1-score.

To understand which detection component contributed the most to the efficacy of our framework, we further conducted an ablation study to remove one component from the system at a time. We used the most challenging evaluation schemes, Iteration-Site, in this ablation test. The results for our three datasets (i.e., Edin, CA, and C-Diff) are depicted in Fig. 4. In general, all three components contributed to the detection correctness, as removing any component would cause decreased precision, recall and F1-score. For Edin and CA, the Auditing component contributed the most (i.e., the detection correctness metrics dropped the most without the Auditing component). On the other hand, for C-Diff the Coefficient component provides the highest contribution to the detection correctness.

### 4.3. Tuned parameters

As shown in Table 2, the β and the γ parameters with the highest Iteration-Site F1-score for the Edin and CA datasets are close, while the ones for the C-Diff dataset are larger. The ranges of the Iteration-Site F1-Score for the Edin, CA and C-Diff datasets are [0.311, 0.423], [0.301, 0.439], and [0.360, 0.421], respectively.

### 4.4. Execution time

**Fig. 5.**
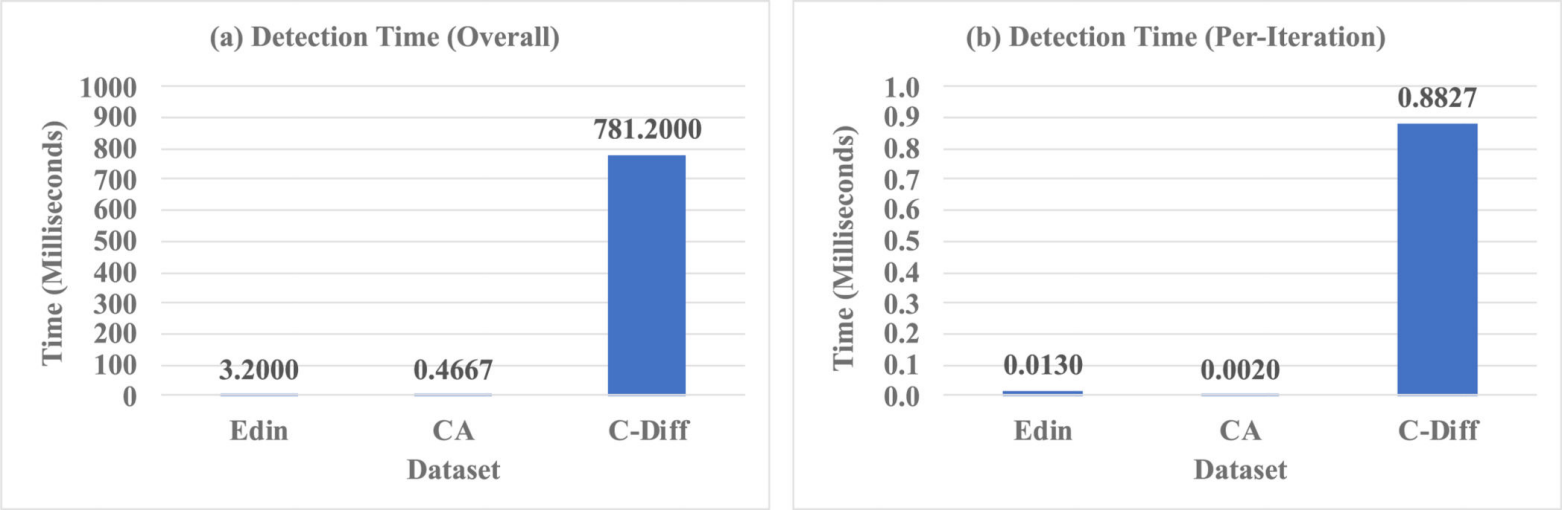
Detection time. **(a)** Overall time computed using all data in the three datasets Edin, CA, and C-Diff under the “all 10 misconduct types” scenario. **(b)** Per-Iteration time, which is the overall time divided by the total number of learning iterations for each dataset.

The detection time results are shown in Fig. 5. The overall time (Fig. 5(a)) is less than one second, and the time required for C-Diff is larger than the other two datasets. The per-iteration time (Fig. 5(b)) demonstrates a similar pattern, and in general the detection can be completed within one millisecond for each learning iteration (the total learning iterations for the Edin, CA, and C-Diff datasets are 246, 238, and 885, respectively).

## 5. Discussion

### 5.1. Findings

In general, our proposed detection method demonstrated good recall (>0.87), which is preferred because the cost of false negatives in the model misconduct scenarios is relatively high. Meanwhile, the precision (>0.74) of the Site-Aggregated scenario shows that our method can also identify misconduct sites with low false positive cases. Identification of misconducts per iteration is harder (the precisions under the Iteration-Aggregated scenario are between 0.62 and 0.75). To precisely detect misconducts for a specific site *and* at a specific iteration is even more challenging (the precisions under the Iteration-Site scenario fall only in [0.20, 0.30]), because of efforts required to identify both the “location” and “time” of the incidence simultaneously. For smaller datasets such as Edin and CA, the Auditing component contributed the most, while for the larger C-Diff dataset, the Coefficient component is more important. Parameter tuning can impact the results (causing at most 0.14 of F1- score change), while the added computational cost is low (<1 ms per iteration).

### 5.2. Limitations

As a proof-of-concept study, the limitations of our work are as follows. (1) *Data*. Although we evaluated our method on three datasets, the evaluation on a dataset with many covariates (e.g., 100 or more) is yet to be conducted. Besides, we are yet to evaluate our method using non-Independent and Identically Distributed (non-IID) settings with different data distributions across sites, with which the detection task could be more challenging. (2) *Model*. The Auditing, Coefficient and Performance components of the framework are readily adoptable; however, the tuning of specific parameters may require additional consideration for generalizability, as well as to bolster the detection success rate in the Iteration-Site scenario. We have yet to investigate the efficacy of the misconduct detection framework on complex machine learning models such as deep neural networks, where some of our assumptions (e.g., the tendency of model coefficients moving towards the same direction) may change. We have also yet to study more complicated prediction tasks such as multi-class or multi-label classifications. (3) *Misconducts*. Our study only considers the situation of applying single misconduct per-iteration-per-site. More exploration needs to be done for multiple misconducts per-iteration-per-site (e.g., applying *both* Gaussian and random noises to the model), or even with more different misconduct types. Besides, the pre-defined misconduct probability values other than the one we used in our experiment (i.e., 0.25) is yet to be evaluated. (4) *Detection*. We tuned the hyper-parameters using simulated model misconduct data, and the tuning process using real-world modeling data warrants investigation. Also, the improvements of precision to reduce false positive rates, as well as the steps after detecting misconducts are yet to be studied.

## 6. Conclusion

We summarized and simulated three categories (plagiarism, fabrication, and falsification) and 10 types of model misconducts under three scenarios (Iteration-Site, Iteration-Aggregated, Site-Aggregated). Then, we developed a detector with three components (Auditing, Coefficient, and Performance) to detect these model misconducts on three datasets (Edin, CA, C-Diff). The results demonstrate desirable high recall, and our detector framework is flexible and extensible to include more detector components to improve its detection capability, and to inform the decision to halt or continue the federated learning process. With the low detection cost of one millisecond per learning iteration, our model misconduct detector framework can enhance the integrity and reliability of federated machine learning on genomic and healthcare data, which in turn contributes to the construction of threat-proof cyber-infrastructure in the medical informatics community.

Although we only conducted experiments on disease-classification machine learning tasks, the misconduct types and detection methods are generalizable. They may be applied to other medical informatics applications such as patient hospitalization [53] and mortality [54]. Moreover, they might also be adopted in a variety of other healthcare-related Information Technology (IT) applications such as insurance [55] and industrial engineering [56], as well as mobile technology [37].

**Table:** Summary Table

What was already known on the topic	Cross-institutional collaborations to use artificial intelligence on clinical/genomic data can accelerate healthcare/genomic medicine research and facilitate quality improvement Real-world risks of incorrect models being submitted to the learning process may reduce the incentives for institutions to participate in the federated modeling consortium
What this study added to our knowledge	• We summarized and simulated three categories and 10 types of model misconducts under three scenarios, and developed a detector framework with three components to detect model misconducts constructed from three datasets
	• The results showed that our method can support the integrity and reliability of federated machine learning on genomic and healthcare data

## Supplementary Material

1

## Figures and Tables

**Table 1:** Model misconduct threat models and adversarial goals. Each type of misconduct can be grouped into one of the three categories: plagiarism (copied model), fabrication (mocked model), and falsification (tampered model), with three possible intentions of hiding information, inspecting information, and disturbing learning.

Threat Models			Adversarial Goals		
	
Category	Type	Number	Hide Info	Inspect Info	Disturb Learning

**Plagiarism**	Self-Plagiarism	#1	Maybe	Yes	Maybe
	Others-Plagiarism	#2	Yes	Yes	Maybe
**Fabrication**	Empty-Fabrication	#3	Yes	Yes	Yes
	Random-Fabrication [7]	#4	Yes	Maybe	Yes
	Gaussian-Fabrication [841]	#5	Yes	Maybe	Yes
**Falsification**	Opposite-Falsification [7]	#6	Yes	Maybe	Yes
	Cosine-Falsification [8]	#7	No	No	Yes
	Random-Falsification [42]	#8	No	No	Yes
	Gaussian-Falsification [47]	#9	No	No	Yes
	Rounded-Falsification	#10	No	No	Yes

**Table 2:** Final parameters tuned using all data in three datasets under the “all types” misconduct scenario. The results of the metrics for each evaluation scheme are also included.

Dataset	Parameter		Iteration-Site			Iteration-Aggregate			Site-Aggregate		
				
	ModelChange Ratio (β)	AUC Difference Threshold (γ)	Precision	Recall	F1-Score	Precision	Recall	F1-Score	Precision	Recall	F1-Score

**Edin**	0.4	0.15	0.273	0.974	0.422	0.728	1.000	0.833	0.875	1.000	0.921
**CA**	0.3	0.15	0.296	0.944	0.438	0.731	0.996	0.827	0.847	0.992	0.887
**C-Diff**	0.9	0.45	0.269	0.983	0.421	0.712	0.998	0.829	1.000	1.000	1.000
