# Supplementary material for: Detecting model misconducts in decentralized healthcare federated learning
Source: Int J Med Inform. Author manuscript; Available in PMC 2023 Jun 9. (PMC10017272; doi:10.1016/j.ijmedinf.2021.104658)
Supplement: 1 [file NIHMS1880390-supplement-1.pdf]

APPENDIX

Detecting Model Misconducts in Decentralized Healthcare Federated Learning

A. DETAILS OF MISCONDUCT GENERATION

A.1 Model Plagiarism

This category of model misconduct includes the types #1 and #2 in **Table 1**. For *self-plagiarism* (#1), the misconduct model (both gradient vector and the variance-covariance matrix) was copied from a model randomly selected from a previous iteration from this site. Similarly, for *others-plagiarism* (#2), the misconduct model was copied from a random previous iteration from a random other site. Note that both plagiarism misconducts were only applied on models not generated in the initialization iteration, so that there would be at least one previous model to be copied from.

A.2 Model Fabrication

This category of misconduct includes the types #3 to #5 in **Table 1**. For *empty-fabrication* (#3), the original model was simply replaced by a zero gradient vector and a zero variance-covariance matrix. For *random-fabrication* (#4), we first identified the maximum and the minimum values of the original gradient vector, and then generated a new gradient vector with values randomly selected between the maximum and the minimum values, to simulate the creation of a vector with more “reasonable” random values. We then adopted the same method to generate the random variance-covariance matrix. For *Gaussian-fabrication* [1] (#5), we first calculated the standard deviation *sd* of the original gradient vector. Then, we created a new gradient vector with values drawn from a Gaussian distribution with zero mean and the standard deviation equals *sd*. Finally, we created an isotropic matrix with *sd* as the new variance-covariance matrix.

A.3 Model Falsification

This category includes the types #6 to #10 in **Table 1**. For *opposite-falsification* [2] (#6), we simply reversed the signs for both gradient vector and variance-covariance matrix. For *cosine-falsification* [1] (#7), we focused on creating a new gradient vector which is similar to the original one, with a given cosine similarity theta (0.63 in our experiment) and the same magnitude as the original gradient vector. For *random-falsification* (#8), we added on each element of the gradient vector a random noise with zero mean and a small-scaled range of absolute value of that element (0.2 in our experiment). A similar process was applied for each element in the variance-covariance matrix to add random noises. Similarly, for *Gaussian-falsification* [3] (#9), we added on each element of the gradient vector and variance-covariance matrix a Gaussian noise with zero mean and a small-scaled range of 0.2 of absolute value of that element. Finally, for *rounded-falsification* (#10), we rounded each element of the gradient vector and variance-covariance matrix to its first non-zero decimal digit (e.g., -110.00761 would be rounded to -110.008).

B. DETAILS OF MISCONDUCT DETECTION

B.1 Auditing Detector

Intuitively, we can simply identify the copied and zero-valued gradient vector or variance-covariance matrix by using the local models recorded on the blockchain ledger. As shown in **Figure 3 (b)**, this detector compares M_{S_T} with the previous local models and sees if M_{S_T} is copied from any of them (including models from site *S* itself), by either duplicating the gradient vector or the variance-covariance matrix. Also, this detector checks to see if M_{S_T} contains an empty gradient vector or variance-covariance matrix. If any of the above situations is detected, model M_{S_T} is considered misconducted.

B.2 Coefficient Detector

The intuition of this detector is that during the model learning iterations, the model coefficients are likely to move towards the same direction until convergence, thus a sudden change in direction may be an indicator for model misconducts. This detector compares the updated model U_{S_T} (computed by updating G_{T-1}, the global model of the previous iteration *T-1*, using only M_{S_T}) with the updated models in the previous iterations (e.g., U_{S_T-1}, U_{S_T-2}, etc.), to recognize any significant direction change (**Figure 3 (c)**). For example, assuming the coefficients in the updated model have been increasing from U_{S_T-2} to U_{S_T-1}, yet a sudden drop from U_{S_T-1} to U_{S_T} may indicate misconduct. In addition, since the model should eventually converge, the enlarged change may also be an indicator of misconduct. Therefore, we consider a direction change or a significant change of a coefficient vector *U* as "the changing of direction or the enlarged change for at least β ratio of the elements in *U*". The changing of direction or the enlarged change of an element is defined as "a change of direction or a larger change difference when compared to the previous iteration". Specifically, suppose *U* [*e*] denotes the eth element of *U*, if (U_{S_T} [*e*] – U_{S_T-1} [*e*]) * (U_{S_T-1} [*e*] – U_{S_T-2} [*e*]) < 0 (i.e., changing of direction) or | U_{S_T} [*e*] – U_{S_T-1} [*e*] | > | U_{S_T-1} [*e*] – U_{S_T-2} [*e*] | (i.e., enlarged change), we consider U_{S_T} [*e*] has a changing of direction or an enlarged change, and thus M_{S_T} would be a potentially misconducted model. On the other hand, β is the *model change ratio*, a parameter ranging from 0 (M_{S_T} is considered misconducted if *any* element of it has changing of direction or enlarged change) to 1 (M_{S_T} is regarded as misconducted only if *all* elements of it have changing of direction or enlarged change).

B.3 Performance Detector

The basic idea of this detector is that during the model learning iterations, the performance of the model (in terms of evaluation results, such as the full Area Under the receiver operating characteristic Curve, or AUC [4 5]) should change gradually, and a significant difference in consecutive evaluation metric values may indicate a potential misconducted model. Thus, as depicted in **Figure 3 (d)**, this detector compares the AUC value of the current updated model U_{S_T} (denoted by AUC(U_{S_T})) with the average of the AUC values of the previous updated models (i.e., AUC(U_{S_1}) to AUC(U_{S_T-1})) and checks if there is a difference with magnitude > γ. The parameter γ, or the *performance difference threshold*, has a range of [0, 0.5]. This is because the AUC values have a range between 0.5 and 1.0, and therefore the maximum possible magnitude of difference is | 1.0 – 0.5 | = 0.5. Specifically, if | AUC(U_{S_T}) – Average (AUC(U_{S_1}), AUC(U_{S_2}), …, AUC(U_{S_T-1})) | > γ, we consider AUC(U_{S_T}) has a significant difference, and thus M_{S_T} would likely be a misconducted model.

B.4 Parameter Tuning

We adopted greedy search (**Figure 3 (e)**) to identify the best values of the two parameters, the model change ratio β and the performance difference threshold γ, for better detection results, based on the grid search method to tune the hyper-parameters of the traditional machine learning algorithms. That is, we used the “training models” (just like “training data”) to identify the best parameters for each dataset, and then evaluated our framework based on the tuned parameters. We then used all data to tune the final parameters, under the scenario that included all 10 types of misconducts.

C. DETAILS OF EXPERIMENT SETTINGS

C.1 Per-Trial 10-Fold Cross-Validation

That is, in each fold, we used the input data from 27 random trials to tune the parameters and used the remaining 3 trials to obtain the evaluation metrics, and the final reported metrics are the average of each fold. The range of β is from 0.0 to 1.0 step 0.1, and the one of γ is from 0.0 to 0.05 step 0.05, resulting 11 * 11 = 121 combinations. The combination with the highest Site-Iteration F1-score was selected as the "best" combination for each fold. The above-mentioned process was repeated for 11 scenarios (i.e., 10 types + 1 "all 10 types" of misconducts). Besides, we also used all 30 trials data to tune a combination of parameters for the "all 10 types" scenario, to identify the final tuned parameters as well as the execution time.

C.2 Implementation

The misconduct generation and detection algorithms were implemented in Java. We adopted GloreChain [6] to create models from the datasets, and leveraged Weka [7] for AUC computation. Our experiments were conducted using the iDASH 2.0 Health Insurance Portability and Accountability Act (HIPAA)-compliant cloud environment [8 9]. The Virtual Machine (VM) on the iDASH 2.0 cloud is based on Linux with the type of Amazon EC2 R5A LARGE (i.e., 2 virtual CPUs and 16GB of RAM) with 100GB of storage.

APPENDIX REFERENCES

1. Kim H, Kim S-H, Hwang JY, Seo C. Efficient privacy-preserving machine learning for blockchain network. IEEE Access 2019; **7**: 136481-95 doi: 10.1109/ACCESS.2019.2940052.
2. Chen X, Ji J, Luo C, Liao W, Li P. When Machine Learning Meets Blockchain: A Decentralized, Privacy-preserving and Secure Design. 2018 IEEE International Conference on Big Data (Big Data); 2018; December 10, 2018 - December 13, 2018. Seattle, WA, United States. IEEE.
3. Blanchard P, El Mhamdi EM, Guerraoui R, Stainer J. Machine learning with adversaries: Byzantine tolerant gradient descent. Proceedings of the 31st International Conference on Neural Information Processing Systems; 2017.
4. Lasko TA, Bhagwat JG, Zou KH, Ohno-Machado L. The use of receiver operating characteristic curves in biomedical informatics. Journal of biomedical informatics 2005; **38** (5): 404-15.
5. Hanley JA, McNeil BJ. The meaning and use of the area under a receiver operating characteristic (ROC) curve. Radiology 1982; **143** (1): 29-36.
6. Kuo T-T, Gabriel RA, Ohno-Machado L. Fair compute loads enabled by blockchain: sharing models by alternating client and server roles. Edited by Suzanne Bakken. Published by Oxford University Press, Kettering, Northants, UK. Journal of the American Medical Informatics Association (JAMIA) 2019; **26** (5): 392-403 doi: 10.1093/jamia/ocy180. March 20, 2019.
7. Hall M, Frank E, Holmes G, Pfahringer B, Reutemann P, Witten IH. The WEKA data mining software: an update. ACM SIGKDD explorations newsletter 2009; **11** (1): 10-18 doi: 10.1145/1656274.1656278.

8. Ohno-Machado L, Bafna V, Boxwala Aa, et al. iDASH. Integrating data for analysis, anonymization, and sharing. Edited by Lucila Ohno-Machado. Published by Oxford University Press, Kettering, Northants, UK. Journal of the American Medical Informatics Association 2012; **19**: 196-201 doi: 10.1136/amiajnl-2011-000538.

9. Ohno-Machado L. To share or not to share: that is not the question. Science translational medicine 2012; **4** (165): 165cm15 doi: 10.1126/scitranslmed.3004454. 2012/12/21.
